# Formononetin alleviated cisplatin-induced acute kidney injury by orchestrating renal tubular cell ferroptosis via PI3K/AKT/NRF2 pathway

**DOI:** 10.3389/fphar.2026.1777367

**Published:** 2026-05-18

**Authors:** Xiang Li, Jiayi Yan, Yao Xu, Zicheng Ling, Tianyi Zhang, Xinghua Shao, Jia Xia, Lumin Tang, Shan Mou

**Affiliations:** 1 Academy of Integrative Medicine, Shanghai University of Traditional Chinese Medicine, Shanghai, China; 2 Molecular Cell Lab for Kidney Disease, Ren Ji Hospital, School of Medicine, Shanghai Jiao Tong University, Shanghai, China; 3 Department of Nephrology, Renji Hospital, School of Medicine, Shanghai Jiao Tong University, Shanghai, China; 4 Punan Branch of Renji Hospital, Shanghai Jiao Tong University School of Medicine (Punan Hospital in Pudong New District, Shanghai), Shanghai, China; 5 Shanghai Jiao Tong University School of Medicine, Shanghai, China

**Keywords:** acute kidney injury, cisplatin nephrotoxicity, ferroptosis, formononetin, Nrf2

## Abstract

**Background:**

Cisplatin chemotherapy is limited by its nephrotoxicity, which leads to acute kidney injury (AKI). Ferroptosis is a key pathogenic mechanism in cisplatin-induced AKI. Formononetin (FN), a natural isoflavone, has demonstrated antioxidant and renoprotective potential, but its role in modulating ferroptosis remains unclear.

**Methods:**

A cisplatin-induced AKI model in mice and a cisplatin-injured HK-2 cell model were established. Kidney injury was assessed via serum creatinine, blood urea nitrogen, H&E staining, and Kim-1 expression. Ferroptosis was evaluated by measuring glutathione (GSH), malondialdehyde (MDA), iron deposition, lipid peroxidation, mitochondrial morphology, and the expression levels of GPX4, SLC7A11, FTH and FTL. The involvement of the NRF2 pathway was investigated using the inhibitor ML385 *in vivo* and siRNA *in vitro*. The role of the PI3K/AKT pathway was assessed using the inhibitor LY294002.

**Results:**

FN treatment significantly improved renal function and attenuated tubular damage in cisplatin-induced AKI. It suppressed key features of ferroptosis, as shown by increased GSH, decreased MDA and iron levels, and preserved mitochondrial integrity. FN also reduced the oxidized lipids, and reversed the cisplatin-induced downregulation of SLC7A11 and GPX4. The renoprotective and anti-ferroptotic effects of FN were abolished by the NRF2 inhibitor ML385 *in vivo* and by siRNA *in vitro*. Furthermore, FN activated the PI3K/AKT pathway, and the PI3K inhibitor LY294002 blocked FN-mediated upregulation of NRF2 and GPX4.

**Conclusion:**

FN protects against cisplatin-induced AKI by inhibiting ferroptosis. This effect is mechanistically associated with the activation of the PI3K/AKT pathway, which subsequently enhances NRF2 signaling and upregulates GPX4 expression. Our findings support FN as a potential candidate for further investigation in the prevention of cisplatin nephrotoxicity.

## 1 Introduction

Acute kidney injury (AKI) is a common and severe clinical syndrome characterized by a rapid decline in renal function, resulting in the accumulation of metabolic waste products and disturbances in fluid and electrolyte homeostasis. Among the diverse etiologies of AKI, cisplatin-induced nephrotoxicity remains one of the most important causes in oncology practice, as it is a major dose-limiting adverse effect of this widely used chemotherapeutic agent for solid tumors such as lung, ovarian, and bladder cancers (Al Suleimani et al., 2025). Cisplatin-associated AKI occurs in approximately 20%–40% of treated patients and substantially restricts the clinical use of cisplatin despite hydration and other supportive strategies (Dasari and Tchounwou, 2014). Because cisplatin is preferentially taken up and accumulated in renal tubular epithelial cells, especially proximal tubules, it induces oxidative stress, mitochondrial dysfunction, inflammatory responses, and multiple forms of regulated cell death, ultimately leading to tubular injury and renal dysfunction (Tang et al., 2023; Elmorsy et al., 2024; Wang et al., 2025). Therefore, identifying effective interventions for cisplatin-induced AKI remains of considerable clinical significance. Prolonged consumption of dietary advanced lipoxidation end products contributes to renal impairment in mice through dysregulated intestinal homeostasis (Wang et al., 2025).

Among the mechanisms implicated in cisplatin-induced AKI, ferroptosis has recently emerged as an important contributor to renal tubular injury (Zhong et al., 2025). Ferroptosis is an iron-dependent form of regulated cell death characterized by excessive lipid peroxidation, reactive oxygen species (ROS) accumulation, and distinct mitochondrial morphological abnormalities (Stockwell et al., 2017). Compared with other forms of cell death, ferroptosis is particularly relevant to renal tubular epithelial cells, which are highly metabolically active and vulnerable to oxidative injury (Hu et al., 2026). A key event in ferroptosis is the collapse of the cellular antioxidant defense system, especially the System Xc^−^–GSH–GPX4 axis. SLC7A11-mediated cystine uptake supports glutathione (GSH) synthesis, while glutathione peroxidase 4 (GPX4) uses GSH to detoxify phospholipid hydroperoxides and maintain membrane integrity. Once this axis is disrupted, lipid peroxides accumulate uncontrollably, leading to membrane damage and cell death (Cao and Dixon, 2016; Lyu et al., 2025; Tsai et al., 2025). Increasing evidence indicates that ferroptosis plays an important role in the pathogenesis of AKI and represents a promising therapeutic target in cisplatin nephrotoxicity (Zhong et al., 2025).

Iron dyshomeostasis is a central driver of ferroptosis because redox-active iron promotes ROS generation through Fenton chemistry and amplifies lipid peroxidation. In renal tubular cells, ferroptotic susceptibility is influenced not only by antioxidant failure but also by dysregulation of iron uptake, storage, utilization, and export. Iron transporters and storage proteins, including transferrin receptor 1 (TFRC/TFR1), and ferroportin, are essential for maintaining intracellular iron balance (Zuo et al., 2024; Wang et al., 2026). Disturbance of this network increases the labile iron pool, enhances oxidative damage, and sensitizes cells to ferroptosis (Hu et al., 2026). In addition, ferritinophagy-mediated ferritin degradation can release ferrous iron (Fe^2+^), thereby further accelerating lipid peroxidation and ferroptotic cell death (Zuo et al., 2024). Therefore, ferroptosis in cisplatin nephrotoxicity is driven by both antioxidant system collapse and iron dysregulation. These observations suggest that both antioxidant system failure and iron dysregulation should be considered when investigating ferroptosis in cisplatin nephrotoxicity.

In the context of cisplatin-induced AKI, cisplatin accumulation in proximal tubular epithelial cells triggers marked oxidative stress and mitochondrial injury, which may interact with iron dysregulation to exacerbate ROS overload and impair the GSH–GPX4 defense system (Cao and Dixon, 2016; Wang et al., 2025). Thus, the interplay between cisplatin accumulation, iron dyshomeostasis, and redox imbalance appears to be a critical pathogenic axis in cisplatin-induced renal injury.

Nuclear factor erythroid 2-related factor 2 (NRF2) is a master regulator of the cellular antioxidant response and a key modulator of ferroptosis. Upon activation, NRF2 induces a broad range of cytoprotective genes involved in antioxidant defense, detoxification, and iron metabolism, including HO-1, SLC7A11, and GPX4-related pathways (Cui et al., 2025; Liu et al., 2026). Through these downstream targets, NRF2 helps preserve redox homeostasis, limit lipid peroxidation, and thereby limiting ferroptosis-associated tissue injury. Therefore, pharmacological strategies that activate NRF2 signaling may represent an effective approach for attenuating cisplatin-induced AKI.

Formononetin (FN), a natural isoflavone mainly isolated from natural plants such as Astragalus membranaceus, has attracted increasing attention because of its anti-inflammatory, antioxidant, and cytoprotective properties (Mao et al., 2025; Wang et al., 2025; Yao et al., 2025). Previous studies have shown that FN exerts renoprotective effects in kidney disease models (Singh et al., 2026). For example, FN improves renal injury in diabetic nephropathy by reducing oxidative stress, metabolic disturbance, and fibrosis (Lv et al., 2020), and alleviates methotrexate-induced nephrotoxicity by suppressing inflammation, apoptosis, and tissue damage (Aladaileh et al., 2019). Furthermore, in a muscle atrophy model associated with chronic kidney disease (CKD), FN improves renal function, nutritional status, and inflammatory markers (Liu, Hu et al., 2021). Although FN has shown efficacy in AKI and CKD models, its underlying mechanisms remain incompletely understood.

In addition, FN has been reported to protect against cisplatin-induced nephrotoxicity through anti-inflammatory and antioxidant mechanisms involving PPARα/NRF2 signaling (Hao et al., 2021), and to attenuate AKI in other settings through modulation of macrophage polarization and NRF2/GPX4-related pathways (Zhang et al., 2024; Zheng et al., 2024). A comprehensive review further highlights its modulation of p38-MAPK, NF-κB, TGF-β, and NRF2/KEAP1 signaling to inhibit pro-inflammatory mediators, enhance antioxidant defenses, and mitigate fibrosis (Singh et al., 2026). However, despite these encouraging findings, it remains unclear whether FN can alleviate cisplatin-induced AKI by suppressing ferroptosis, particularly through NRF2-dependent regulation of ferroptosis. Therefore, in the present study, we investigated the protective effect of FN against cisplatin-induced AKI *in vivo* and *in vitro*, with particular emphasis on ferroptosis-related injury and NRF2-associated signaling.

## 2 Materials and methods

### 2.1 Reagents and antibodies

Cisplatin (HY-17394), formononetin (HY-N0183), LY294002 (HY-10108), Deferoxamine (DFO) (HY-B0988) and ML385 (HY-100523) were purchased from MedChemExpress. Anti-GPX4 (67763-1-Ig; 1:1000) antibody was purchased from Proteintech. Anti-SLC7A11 (DF12509; 1:1000) antibody was purchased from Affinity Biosciences. Anti-FTH (TD6278; 1:1000) and anti-FTL (T56955; 1:1000) antibodies were purchased from Abmart. Anti-PI3K (ab86714; 1:1000) antibody was purchased from Abcam. Anti-Kim-1 (AF 1817, 1:1000) antibody was purchased from R&D Systems. Anti-p-PI3K (4228; 1:1000), anti-p-AKT (4060; 1:1000), anti-AKT (9272; 1:500), anti-Heme Oxygenase-1 (HO-1) (26416; 1:1000) and anti-NRF2 (12721; 1:1000) antibodies were purchased from Cell Signaling Technology. Anti-β-actin (AF0003; 1:10000) or anti-GAPDH (AF0006; 1:10000) antibodies were purchased from Beyotime Biotechnology.

### 2.2 Animals

Male C57BL/6 mice (4–5 weeks old) weighing 20–22 g were obtained from the Slac Laboratory Animal Center. Mice were housed under standard laboratory conditions. Food and water were provided *ad libitum*. All animal procedures were conducted in accordance with the Animal Protocol Committee of Shanghai Jiaotong University and were approved by the Animal Care Committee at Renji Hospital, School of Medicine, Shanghai Jiao Tong University.

### 2.3 Animal experiments

**FIGURE 1:**
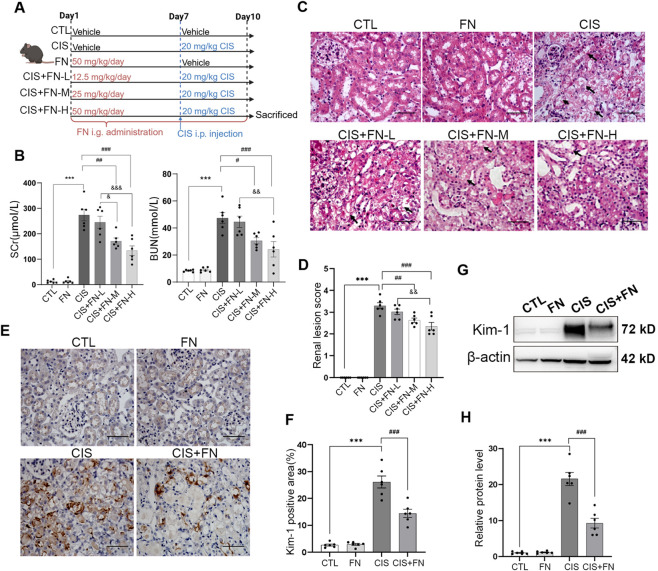
FN Alleviated Renal Injury in Mice with Cisplatin-Induced AKI. **(A)** Schematic diagram of the cisplatin-induced acute kidney injury mouse model and formononetin administration (created with BioRender); **(B)** SCr and BUN levels in each group of mice; **(C)** Representative images of H&E-stained renal tissue sections (scale bar, 100 μm); black arrows indicate damaged tubules; **(D)** Quantification of renal lesion score; **(E)** Representative immunohistochemical staining of Kim-1 in renal tissues (scale bar, 100 μm); **(F)** Quantification of the Kim-1-positive area shown in **(E)**; **(G)** Western blot analysis of Kim-1 protein expression in renal tissues; **(H)** Densitometric quantification of Kim-1 protein levels shown in panel G. ***P < 0.001; ^#^P < 0.05, ^##^P < 0.01, ^###^P < 0.001; ^&^P < 0.05, ^&&^P < 0.01, ^&&&^P < 0.001 vs. indicated group. CTL, control; CIS, cisplatin-induced AKI (20 mg/kg); FN, formononetin alone (50 mg/kg); CIS + FN-L, cisplatin-induced AKI mice treated with low-dose FN (12.5 mg/kg); CIS + FN-M, cisplatin-induced AKI mice treated with medium-dose FN (25 mg/kg); CIS + FN-H, cisplatin-induced AKI mice treated with high-dose FN (50 mg/kg); SCr, Serum creatinine; BUN, Blood urea nitrogen.

To evaluate the effects of FN against cisplatin-induced AKI, mice were randomly divided into six groups after 1 week of acclimatization, including an untreated control group (CTL), cisplatin-induced AKI group (CIS, 20 mg/kg), FN alone treatment group (FN, 50 mg/kg), cisplatin-induced AKI mice treated with low-, medium-, and high-dose of FN (CIS + FN-L, 12.5 mg/kg; CIS + FN-M, 25 mg/kg; CIS + FN-H, 50 mg/kg). FN was dissolved in DMSO/corn oil (9/1, v/v). We selected 12.5, 25, and 50 mg/kg/day of FN administered by intragastric gavage as the treatment doses. FN was administered daily by intragastric gavage for ten consecutive days, before which blood and tissues were harvested. Cisplatin (20 mg/kg) was administered via intraperitoneal injection on day 7 to induce AKI (Figure 1A). To verify whether FN exerts protective effects against AKI through NRF2 activation, 30 mg/kg/day of ML385 was administered once daily from days 8–10 via intraperitoneal injection, 2 h before FN treatment (ML385 group). The doses of cisplatin (Lin et al., 2023), FN (Zhou et al., 2023; Zhu et al., 2023; Zhang et al., 2024) and ML385 (Li et al., 2022) were selected based on previous studies. Blood was withdrawn from the abdominal aorta under light anesthesia induced by sodium pentobarbital (10 mg/kg, *i. p.*) for the determination of serum kidney function parameters. Following blood collection, the mice were euthanized via decapitation, and their kidneys were promptly excised and decapsulated. Briefly, each kidney was divided into two-halves along the longitudinal axis. One portion of the kidney was fixed in formalin for histopathological analyses, including hematoxylin and eosin (H&E) staining and immunohistochemistry. Separate renal cortex samples were processed for transmission electron microscopy. The remaining half was trimmed to remove perirenal fat and the pelvis/ureteral portion, cut into small pieces, snap-frozen in liquid nitrogen, and stored at −80 °C for subsequent biochemical analyses, including Western blot and oxidative stress-related assays.

### 2.4 Cell culture and treatments

The human proximal tubular cell line (HK-2) was obtained from the American Type Culture Collection and cultured in Dulbecco’s Modified Eagle Medium/Nutrient Mixture F-12 (DMEM/F-12) (BasalMedia, L310KJ) supplemented with 10% fetal bovine serum (FBS, Excell, FSP500) at 37 °C in a 5% CO_2_ humidified incubator. *Mycoplasma* contamination was excluded using a MycoColor one-step *mycoplasma* detection assay (Vazyme, D201-01). For cell viability assays, cells were seeded in 96-well plates at an initial density of 8,000 cells per well; for Western blot assays, cells were seeded in 60-mm-dishes at 8 × 10^5^ cells per dish. According to prior reports (Lin et al., 2023), after 12 h of attachment (>80% confluence), cells were treated with 20 μmol/L cisplatin or other agents under reduced serum conditions (2% FBS) for 24 h. Unless otherwise specified, cells were pretreated with FN for 24 h followed by cisplatin exposure for an additional 24 h. Cell viability was detected by CCK-8 assay according to the manufacturer’s instructions (APExBIO, K1018). The absorbance was measured at OD_450_ using a microplate reader (BioTek).

### 2.5 Renal function

SCr and BUN concentrations were measured using commercially available colorimetric assays, obtained from Nanjing Jiancheng (C011-2-1 and C013-2–1).

### 2.6 Western blot

Total proteins were extracted from tissues or cells using RIPA lysis buffer (Solarbio, R0010), supplemented with phosphatase inhibitor cocktail (Roche, 4906837001), and protease inhibitor cocktail (Roche, 4693132001). Protein concentrations were quantified with a BCA assay kit (Beyotime, P0010S). Equal amounts of protein were separated by sodium dodecyl sulfate-polyacrylamide gel electrophoresis and transferred onto polyvinylidene fluoride membranes (PVDF, Millipore, IPVH00005). The membranes were blocked with 5% non-fat milk in Tris-buffered saline containing Tween-20 (TBST) for 1–2 h at room temperature. Subsequently, they were incubated overnight at 4 °C with specific primary antibodies. After washing with TBST, the membranes were probed with horseradish peroxidase-conjugated secondary antibodies (Beyotime, A0277 and A0192) at room temperature. Protein bands were visualized using enhanced chemiluminescence substrate, and band intensities were quantified by densitometry with ImageJ software (V 1.50b). β-actin or GAPDH antibodies were used as internal controls for normalization.

### 2.7 Histopathological examination

The fixed renal specimens were dehydrated in a graded ethanol series, cleared with xylene, and embedded in paraffin wax. Subsequently, 5-μm-thick serial sections were cut with a rotary microtome and mounted onto glass slides for histological analysis. For histological evaluation, these sections were subjected to H&E staining. The stained sections were examined by an experienced histopathologist in a blinded manner using a high-definition microscopic imaging system (Leica Microsystems GmbH). Tubular damage was evaluated using renal lesion score according to a previously reported scoring system (Xia et al., 2025). For electron microscopy, renal cortex tissues were rapidly dissected into 1 × 1 × 3 mm cubes at 4 °C and immediately fixed in electron microscopy fixative overnight at 4 °C in the dark. The specimens were then dehydrated through a graded ethanol series containing 3% uranyl acetate, infiltrated with a mixture of epoxy resin and propylene oxide overnight, and subsequently polymerized. Ultrathin sections (70 nm in thickness) were obtained, contrasted with lead citrate, and visualized under an HT-7800 transmission electron microscope (Hitachi) according to the previous study (Lin et al., 2019).

### 2.8 GSH detection

Reduced GSH levels were evaluated using a colorimetric assay (Nanjing Jiancheng, A006-2-1). Briefly, kidney tissues were homogenized, centrifuged, and the resulting supernatants were used for GSH detection. The content of GSH was measured using a colorimetric method based on the reaction with 5,5′-dithiobis-2-nitrobenzoic acid, which produces a yellow compound that can be quantified by measuring the absorbance at OD_405_.

### 2.9 Malondialdehyde (MDA) detection

MDA content was evaluated using a lipid peroxidation assay (Nanjing Jiancheng, A003-1–2). Treated cells or kidney tissues were lysed and centrifuged, and the supernatant was heated with thiobarbituric acid. Absorbance was measured at OD_532_.

### 2.10 Iron deposition detection

Iron deposition in renal tissues was detected using Prussian blue staining with a Prussian Blue Staining Kit (DAB Enhancement Method) (Solarbio, G1428), according to the manufacturer’s instructions. After Prussian blue staining followed by DAB enhancement, iron-positive signals in the tissue sections appeared yellow-brown.

### 2.11 ROS determination

Cellular ROS production was detected using DCFH-DA assay (Beyotime Biotechnology, S0033S). Treated HK-2 cells in 6-well plates were incubated with 10 μmol/L DCFH-DA in serum-free DMEM at 37 °C for 30 min in the dark. After washing twice with serum-free DMEM, cells were collected and subjected to flow cytometric analysis.

### 2.12 Intracellular iron detection

Intracellular Fe^2+^ levels in HK-2 cells were assessed using the FerroOrange fluorescent probe (1 μmol/L; Dojindo, F374) according to the manufacturer’s instructions. Fluorescence images were captured under a fluorescence microscope and quantified using ImageJ.

### 2.13 Detection of lipid peroxidation

Lipid peroxidation in HK-2 cells was evaluated using the BODIPY 581/591 C11 fluorescent probe (10 μmol/L; Dojindo, L267) according to the manufacturer’s instructions. This probe distinguishes oxidized and non-oxidized lipids, with red fluorescence indicating the non-oxidized form and green fluorescence indicating the oxidized form. Fluorescence images were captured under a fluorescence microscope and quantified using ImageJ.

### 2.14 siRNA transfection

Prior to transfection, HK-2 cells were seeded in 6-well plates and cultured overnight. The cells were then transfected with 50 nM of either siRNA targeting *NRF2* (si*NRF2*) or a negative control siRNA (siNC), using Lipofectamine RNAiMAX transfection reagent (Invitrogen, 13778030) according to the manufacturer’s protocol. SiRNA sequences were as follows: si*NRF2* sense, 5ʹ-CCC​UGU​UGA​UUU​AGA​CGG​UAU​tt-3ʹ, and antisense, 5ʹ-AUA​CCG​UCU​AAA​UCA​ACA​GGG​tt-3ʹ. After 72 h of incubation, cells were harvested, and transfection efficiency was evaluated by measuring NRF2 protein levels via Western blot analysis.

### 2.15 Statistical analysis

The sample size for *in vivo* animal experiments was calculated using G_Power 3.1.9.7 based on our previous study using cisplatin-induced AKI model (Lin et al., 2023). Briefly, n = 6 for *in vivo* animal experiments, and n = 3–6 for *in vitro* cell experiments. The results were expressed as mean ± standard error of the mean. For comparisons among multiple groups, one-way analysis of variance was performed, followed by Tukey’s *post hoc* test. For comparisons between two groups, the Student’s t-test was used for data conforming to a normal distribution, while the non-parametric Mann-Whitney U test was applied for data that did not. All statistical analyses were conducted using GraphPad Prism version 9, and a p-value of less than 0.05 was considered statistically significant.

## 3 Results

### 3.1 FN Alleviated renal injury in mice with cisplatin-induced AKI

We first evaluated the effect of FN in a mouse model of cisplatin-induced AKI (Figure 1A). Cisplatin challenge markedly increased SCr and BUN levels compared to the control group (Figure 1B), confirming successful AKI induction. FN alone had no effect on these parameters. Conversely, FN treatment attenuated the cisplatin-induced elevations in SCr and BUN in a dose-dependent manner, with significant improvements observed in the medium dose (CIS + FN-M) and high dose (CIS + FN-H) groups. These findings indicate that FN effectively protects against cisplatin-induced renal dysfunction. Histopathological assessment by H&E staining revealed that cisplatin injection induced severe renal damage, characterized by tubular epithelial cell swelling, and necrosis. These injuries were attenuated by medium and high doses of FN (Figures 1C,D). Because the high dose showed the strongest efficacy without observable toxicity, it was selected for subsequent mechanistic experiments. We next assessed the expression of the renal injury marker Kim-1 by both immunohistochemistry (IHC) (Figures 1E,F) and Western blot (Figures 1G,H). Both results showed that compared with the control group, Kim-1 expression was significantly upregulated in the cisplatin group. In contrast, Kim-1 levels in the FN group were comparable to those in the control group (P > 0.05). Notably, FN treatment markedly attenuated the cisplatin-induced elevation of Kim-1, showing a significant reduction compared to the cisplatin group (Figures 1F,H).

### 3.2 FN Mitigated Ferroptosis in Cisplatin-Induced AKI mice

**FIGURE 2:**
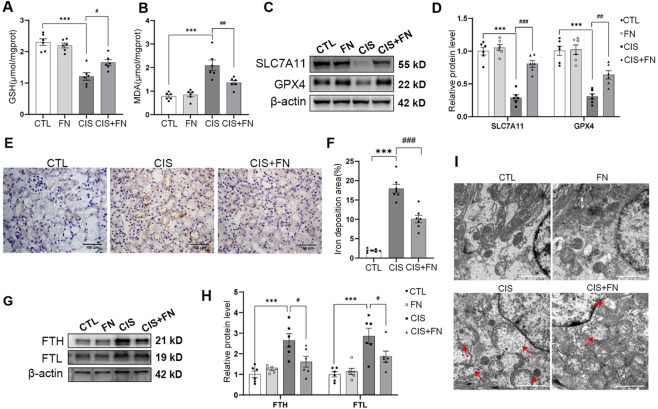
FN Mitigated Ferroptosis in Cisplatin-Induced AKI Mice **(A)** Renal GSH levels in each group; **(B)** Renal MDA levels in each group of mice; **(C)** Western blot analysis of SLC7A11 and GPX4 protein expression; **(D)** Densitometric quantification of SLC7A11 and GPX4 protein levels shown in **(C)**; **(E)** Representative images of renal iron deposition in each group; **(F)** Quantification of iron-positive areas shown in **(E)**; **(G)** Western blot analysis of FTL and FTH protein expression in renal tissues; **(H)** Densitometric quantification of FTL and FTH protein levels shown in **(G)**; **(I)** Representative transmission electron microscopy images of mitochondrial morphology in renal tissues (scale bar, 2 μm). ^***^P < 0.001; ^#^P < 0.05, ^##^P < 0.01, ^###^P < 0.001 vs. indicated group. CTL, control; CIS, cisplatin-induced AKI (20 mg/kg); FN, formononetin alone (50 mg/kg); CIS + FN, cisplatin-induced AKI mice treated with FN (50 mg/kg); GSH, glutathione; MDA, malondialdehyde. For mechanistic analyses, the high-dose FN group (50 mg/kg) was used and is indicated as CIS + FN.

To determine whether the protective effect of FN involved inhibition of ferroptosis, we assessed hallmarks of this process, including iron metabolism and lipid peroxides. We assessed ferroptosis-related oxidative stress by measuring GSH and MDA levels (Figures 2A,B). The cisplatin group displayed a profile indicative of pronounced oxidative stress, characterized by significantly reduced GSH and elevated MDA. Treatment with FN reversed these alterations, indicating its efficacy in attenuating cisplatin-induced lipid peroxidation. The SLC7A11/GSH/GPX4 axis is a core antioxidant system that suppresses lipid peroxidation during ferroptosis. Inhibition of SLC7A11 leads to reduced synthesis of the major antioxidant GSH, which subsequently diminishes GPX4 activity. Western blot analysis revealed that cisplatin challenge significantly decreased the protein levels of both SLC7A11 and GPX4. Treatment with FN significantly rescued the cisplatin-induced reduction of both SLC7A11 and GPX4 proteins (Figures 2C,D). Given the central role of iron metabolism in ferroptosis, we further evaluated renal iron deposition and ferritin complex components (FTH and FTL) in cisplatin-induced AKI. DAB-enhanced Prussian blue staining revealed prominent iron deposition after cisplatin challenge, while the FN treatment reduced renal deposition (Figures 2E,F). Western blot analysis revealed a significant upregulation of both FTL and FTH in the cisplatin group. Treatment with FN significantly downregulated the cisplatin-induced overexpression of FTL and FTH (Figures 2G,H). Mitochondrial dysfunction is a hallmark of ferroptosis. Electron microscopy revealed significant mitochondrial damage in the cisplatin group, including ruptured mitochondria, diminished cristae, and a shorter, more rounded morphology. Treatment with FN significantly ameliorated these pathological changes (Figure 2I). Taken together, these results indicate that FN attenuated ferroptosis-associated changes in cisplatin-induced AKI mice.

### 3.3 FN ameliorated ferroptosis in cisplatin-induced AKI by upregulating NRF2 expression

**FIGURE 3:**
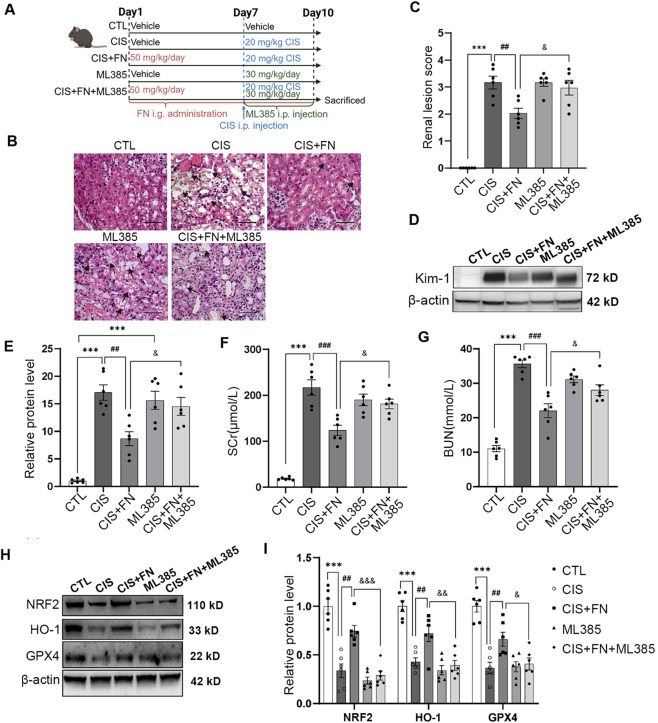
FN Ameliorated Ferroptosis in Cisplatin-Induced AKI by Upregulating NRF2 Expression **(A)** Schematic diagram of the cisplatin-induced acute kidney injury mouse model and treatment with formononetin and/or ML385 (created with BioRender); **(B)** Representative images of H&E-stained kidney sections from mice treated with FN and/or ML385 (scale bar, 100 μm); black arrows indicate injured tubules; **(C)** Quantification of renal lesion scores based on **(B)**; **(D)** Western blot analysis of Kim-1 protein expression in renal tissues from mice treated with FN and/or ML385; **(E)** Densitometric quantification of Kim-1 protein levels shown in **(D)**; **(F)** SCr levels in each group; **(G)** BUN levels in each group; **(H)** Western blot analysis of NRF2, HO-1, and GPX4 protein expression in renal tissues from mice treated with FN and/or ML385; **(I)** Densitometric quantification of NRF2, HO-1, and GPX4 protein levels shown in panel H. ^***^P < 0.001; ^##^P < 0.01, ^###^P < 0.001; ^&^P < 0.05 vs. indicated group. CTL, control; CIS, cisplatin-induced AKI (20 mg/kg); CIS + FN, cisplatin-induced AKI mice treated with FN (50 mg/kg); ML385, mice treated with ML385 alone (30 mg/kg); CIS + FN + ML385, cisplatin-induced AKI mice treated with FN and ML385; SCr, Serum creatinine; BUN, Blood urea nitrogen.

Because changes in FTH and FTL may represent secondary responses to iron dysregulation, we next focused on the SLC7A11/GPX4 system as a potential upstream regulator of ferroptosis in cisplatin-induced kidney injury. To further investigate whether FN protects against cisplatin-induced AKI by regulating NRF2 to inhibit ferroptosis, ML385 (30 mg/kg), a pharmacological inhibitor of NRF2, was administered via intraperitoneal injection to suppress NRF2 protein expression (Figure 3A). To determine whether the renoprotective effect of FN was NRF2-dependent, we evaluated key injury parameters under NRF2 suppression with ML385. ML385 effectively abolished the beneficial effects of FN. Specifically, it attenuated FN-mediated improvements in histopathological injury (H&E staining and Kim-1 expression, Figures 3B–E) and renal function (SCr and BUN, Figures 3F,G), confirming the functional involvement of NRF2. Mechanistically, ML385 significantly suppressed NRF2 protein levels. Furthermore, ML385 treatment counteracted the FN-induced upregulation of the NRF2 and the downstream targets HO-1 and GPX4 in mice with cisplatin-induced AKI (Figures 3H,I)**.** Based on the observed renoprotective effects and potential mechanisms of FN in the mouse AKI model, we further investigated its impact on ferroptosis using a cisplatin-induced injury model in human HK-2 cells.

### 3.4 FN alleviated cisplatin-induced ferroptosis in HK-2 cells

**FIGURE 4:**
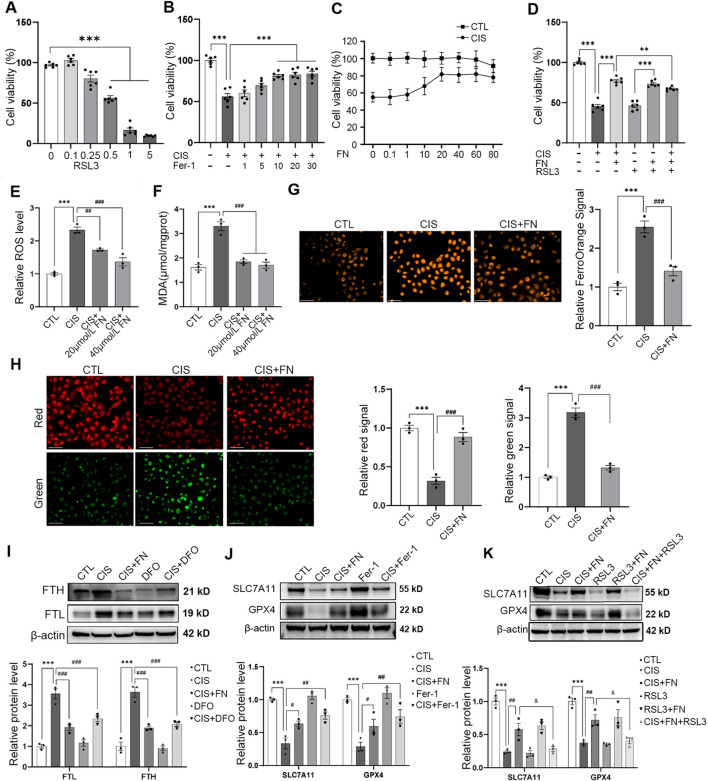
FN Alleviated Cisplatin-Induced Ferroptosis in HK-2 Cells **(A)** Cell viability was assessed by CCK-8 assay after treatment with increasing concentrations of RSL3 for 24 h; **(B)** Cell viability was measured after pretreatment with increasing concentrations of Fer-1 for 24 h followed by 20 μmol/L cisplatin for an additional 24 h; **(C)** Cell viability was measured after pretreatment with increasing concentrations of FN for 24 h followed by 20 μmol/L cisplatin for an additional 24 h; **(D)** Cell viability was assessed after pretreatment with 40 μmol/L FN for 24 h followed by 20 μmol/L cisplatin or 0.5 μmol/L RSL3 for an additional 24 h; **(E)** Intracellular ROS levels were measured using the DCFH-DA fluorescent probe after FN pretreatment followed by cisplatin exposure; **(F)** Cellular MDA levels were measured after FN pretreatment followed by cisplatin exposure; **(G)** Intracellular Fe^2+^ levels in HK-2 cells were assessed using the FerroOrange fluorescent probe. Left, representative fluorescence images (scale bar, 100 μm); right, quantitative analysis of intracellular Fe^2+^ levels; **(H)** Lipid peroxidation in HK-2 cells was evaluated using the BODIPY 581/591 C11 fluorescent probe. Left, representative fluorescence images (scale bar, 100 μm). Middle, quantitative analysis of red-labeled non-oxidized lipids. Right, quantitative analysis of green-labeled oxidized lipids; **(I)** Western blot analysis of FTL and FTH protein expression after pretreatment with 40 μmol/L FN or 10 μmol/L DFO for 24 h followed by cisplatin for an additional 24 h. Top, representative immunoblots; bottom, densitometric quantification of FTL and FTH protein levels; **(J)** Western blot analysis of SLC7A11 and GPX4 protein expression after pretreatment with 40 μmol/L FN or 10 μmol/L Fer-1 for 24 h followed by cisplatin for an additional 24 h. Top, representative immunoblots; bottom, densitometric quantification of SLC7A11 and GPX4 protein levels; **(K)** Western blot analysis of SLC7A11 and GPX4 protein expression after pretreatment with 40 μmol/L FN for 24 h followed by 20 μmol/L cisplatin or 0.5 μmol/L RSL3 for an additional 24 h. Top, representative immunoblots; bottom, densitometric quantification of SLC7A11 and GPX4 protein levels. ^***^P < 0.001; ^#^P < 0.05, ^##^P < 0.01, ^###^P < 0.001; ^&^P < 0.05 vs. indicated group. CTL, control; CIS, HK-2 cells treated with 20 μmol/L cisplatin; CIS + FN, HK-2 cells treated with cisplatin plus 40 μmol/L FN; RSL3, HK-2 cells treated with 0.5 μmol/L RSL3; RSL3+FN, HK-2 cells treated with RSL3 plus 40 μmol/L FN; CIS + FN + RSL3, HK-2 cells treated with cisplatin, FN, and RSL3; Fer-1, ferrostatin-1; ROS, reactive oxygen species; MDA, malondialdehyde.

We used Ras-Selective Lethal 3 (RSL3), a known ferroptosis inducer, to directly trigger ferroptosis. RSL3 reduced cell viability in a concentration-dependent manner (Figure 4A). A concentration of 0.5 μmol/L, which decreased viability to approximately 60%, was selected for subsequent experiments. The involvement of ferroptosis was first supported by the protective effect of ferrostatin-1 (Fer-1) against cisplatin-induced cell death (Figure 4B). Treatment with FN significantly restored the viability of cells injured by either CIS or RSL3 (Figures 4C,D). This consistent protective effect against both agents suggests that FN may, at least in part, counteract ferroptosis-related cell injury. FN co-treatment significantly suppressed the cisplatin-induced elevations in both ROS and MDA levels (Figures 4E,F). Furthermore, FerroOrange and BODIPY 581/591 C11 probes were applied after cisplatin incubation to detect iron metabolism and lipid peroxidation.

Cisplatin increased intracellular Fe2^+^ accumulation and oxidized lipid levels. Both intracellular Fe^2+^ accumulation and lipid peroxidation induced by cisplatin were markedly attenuated by FN (Figures 4G,H). Concurrently, FN also downregulated the cisplatin-induced ferritin subunits FTH and FTL upregulation, suggesting that FN modulates cellular iron metabolism, with effects comparable to those observed for the iron chelator DFO under the present conditions (Figure 4I). Furthermore, FN significantly counteracted the cisplatin-induced suppression of GPX4 and SLC7A11, similar to the known inhibitor Fer-1 (Figure 4J). The ability of FN to restore these proteins was also observed in the presence of both cisplatin and the ferroptosis inducer RSL3, although RSL3 partially reversed the effect of FN (Figure 4K). These findings collectively suggest that FN acts as an inhibitor of ferroptosis with cytoprotective functions in cisplatin-induced injury.

### 3.5 FN inhibited ferroptosis by regulating the PI3K/AKT/NRF2 pathway

**FIGURE 5:**
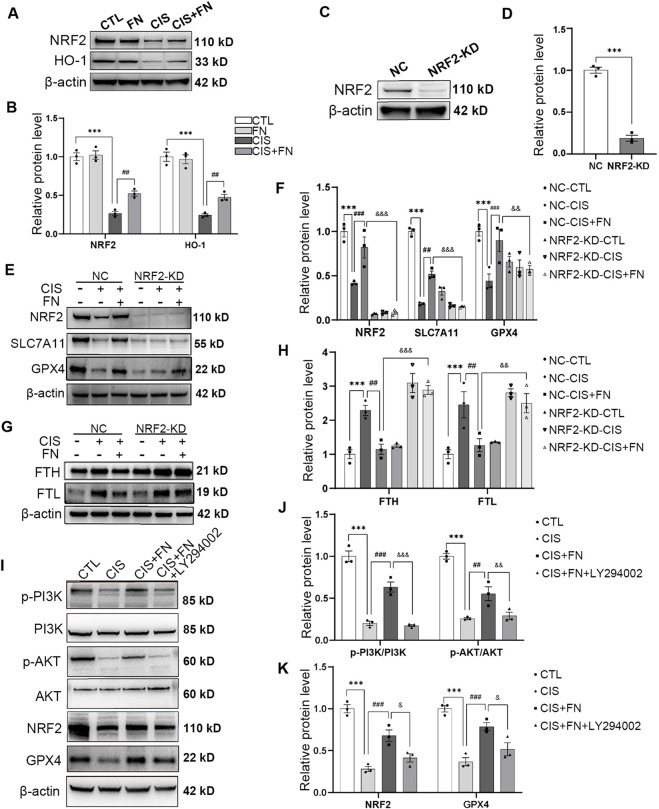
FN Inhibited Ferroptosis by Regulating the PI3K/AKT/NRF2 Pathway **(A)** Representative immunoblots of NRF2 and HO-1 protein expression in HK-2 cells pretreated with 40 μmol/L FN for 24 h followed by 20 μmol/L cisplatin for an additional 24 h; **(B)** Densitometric quantification of NRF2 and HO-1 protein levels shown in **(A)**; **(C)** Evaluation of NRF2 knockdown efficiency by Western blot after transient transfection with si*NRF2*; **(D)** Densitometric quantification of NRF2 protein expression in NC and *NRF2*-knockdown HK-2 cells shown in **(C)**; **(E)** Western blot analysis of NRF2, SLC7A11, and GPX4 protein expression in NC and *NRF2*-knockdown HK-2 cells treated with 40 μmol/L formononetin for 24 h followed by 20 μmol/L cisplatin for an additional 24 h; **(F)** Densitometric quantification of NRF2, SLC7A11, and GPX4 protein levels shown in **(E)**; **(G)** Western blot analysis of FTL and FTH protein expression in NC and NRF2-knockdown HK-2 cells under the same treatment conditions as in **(E)**; **(H)** Densitometric quantification of FTL and FTH protein levels shown in **(G)**; **(I)** Western blot analysis of p-PI3K/PI3K, p-AKT/AKT, NRF2, and GPX4 protein expression in HK-2 cells treated with 20 μmol/L LY294002 and/or 40 μmol/L formononetin for 24 h followed by 20 μmol/L cisplatin for an additional 24 h; **(J)** Quantitative analysis of the p-PI3K/PI3K and p-AKT/AKT ratios shown in **(I)**; **(K)** Quantitative analysis of NRF2 and GPX4 protein levels shown in **(I)**. ^**^P < 0.01, ^***^P < 0.001; ^#^P < 0.05, ^##^P < 0.01, ^###^P < 0.001; ^&^P < 0.05 vs. indicated group. CTL, control; CIS, HK-2 cells treated with 20 μmol/L cisplatin; FN, HK-2 cells treated with 40 μmol/L FN alone; CIS + FN, HK-2 cells treated with cisplatin plus 40 μmol/L FN; CIS + FN + LY294002, HK-2 cells treated with cisplatin, 40 μmol/L FN, and 20 μmol/L LY294002; NC, Negative control; *NRF2*-KD, *NRF2*-knockdown.

To further elucidate the mechanism by which FN inhibits ferroptosis, we examined the protein levels of key components of the PI3K/AKT/NRF2 signaling pathway *in vitro*. Exposure to cisplatin significantly reduced the levels of NRF2 and its downstream target HO-1. This effect was reversed by FN, which restored their expression to near-normal levels, whereas FN alone had no significant effect, indicating that FN specifically counteracts cisplatin-induced suppression of the NRF2/HO-1 axis (Figures 5A,B). To establish a causal link between NRF2 and FN’s action, we employed an *NRF2*-knockdown model (Figures 5C,D). Silencing NRF2 expression effectively abolished the protective effects of FN across multiple ferroptosis-related endpoints. In control cells, FN restored the expression of SLC7A11 and GPX4 that were suppressed by cisplatin, and concurrently inhibited cisplatin-induced FTH and FTL upregulation. However, in NRF2-deficient cells, FN largely lost its ability to modulate these key proteins. The dependence of FN’s effects on NRF2 supports NRF2 as a key mediator of FN’s inhibition of ferroptosis and regulation of iron metabolism in cisplatin-injured HK-2 cells (Figures 5E–H). To investigate whether FN upregulates NRF2 by activating the upstream PI3K/AKT pathway, we treated HK-2 cells with LY294002, a specific PI3K inhibitor. The results demonstrated that FN effectively rescued the cisplatin-induced suppression of the p-PI3K/PI3K and p-AKT/AKT ratios, as well as the protein levels of NRF2 and GPX4. However, these effects of FN were markedly attenuated in the presence of LY294002 (Figures 5I–K). These findings suggest that FN enhance the expression of NRF2 and GPX4, at least in part, by activating PI3K/AKT phosphorylation, thereby inhibiting ferroptosis.

## 4 Discussion

Ferroptosis is an iron-dependent form of regulated cell death that is distinct from apoptosis, necrosis, autophagy, and pyroptosis in its morphological, biochemical, and genetic features (Cao and Dixon, 2016). It is characterized by iron overload, excessive ROS production, and the accumulation of lipid peroxidation products (Trujillo-Alonso et al., 2019). Because renal tubular epithelial cells are highly active in iron handling and redox regulation, the kidney is particularly susceptible to ferroptotic injury (Scindia Ph et al., 2019). In recent years, ferroptosis has been increasingly recognized as an important mechanism in a wide range of kidney diseases, including AKI, CKD, and renal cell carcinoma (Green et al., 2022; Wang et al., 2022; Zhang et al., 2023). In the present study, we found that FN markedly attenuated cisplatin-induced renal injury *in vivo* and *in vitro*, and this protection was accompanied by suppression of ferroptosis-related changes, restoration of antioxidant capacity, and activation of PI3K/AKT/NRF2 signaling.

Growing evidence suggests that ferroptosis is not only involved in the pathogenesis of AKI but also represents a promising therapeutic target. Previous studies have shown that pharmacological inhibition of ferroptosis can ameliorate renal injury in multiple AKI models. For instance, thiazolidinedione drugs, such as troglitazone and pioglitazone, have been shown to specifically inhibit acyl-CoA synthetase long-chain family member 4 (ACSL4), thereby reducing the accumulation of lipid peroxidation products induced by ferroptosis activators and protecting against ferroptosis, offering new perspectives for AKI treatment (Wang et al., 2025). Activation of the vitamin D receptor increased GPX4 expression and suppressed ferroptosis in cisplatin-induced AKI (Hu et al., 2020). Whereas ferrostatin-1 significantly improved renal function in glycerol-induced rhabdomyolysis-associated AKI (Guerrero-Hue et al., 2019). Consistent with these reports, our *in vitro* data showed that ferrostatin-1 improved the viability of cisplatin-treated HK-2 cells and restored SLC7A11 and GPX4 expression, further supporting the involvement of ferroptosis in cisplatin-induced tubular injury. More importantly, FN exerted similar protective effects, suggesting that suppression of ferroptosis is an important component of its renoprotective action.

Iron homeostasis is tightly controlled by multiple proteins, and its disruption is a central event in ferroptosis (Hentze et al., 2010). Ferritin, composed of ferritin heavy chain (FTH) and ferritin light chain (FTL), serves as a major intracellular iron storage complex and limits iron-mediated oxidative injury (Scindia Ph et al., 2019). In kidney disease, alterations in ferritin expression may reflect adaptive or pathological responses to disturbed iron metabolism. For example, proximal tubule-specific deletion of FTH aggravates rhabdomyolysis-induced AKI, highlighting its protective role against iron-dependent injury (Zarjou et al., 2013). On the other hand, increased ferritin expression has also been observed in cisplatin-induced AKI and sepsis-associated kidney injury, possibly reflecting compensatory responses to oxidative stress and iron overload (Sharma and Leaf, 2019; Guo et al., 2022). Iron chelators like DFO have been shown to prevent AKI in various models (Sharma and Leaf, 2019). In the present study, cisplatin increased the expression of ferritin subunits, whereas FN partially normalized these changes. These findings suggest that FN may alleviate cisplatin-induced iron dysregulation and contribute to the restoration of intracellular iron homeostasis. However, these data should be interpreted cautiously, as changes in ferritin expression and iron amount alone do not directly prove ferroptosis inhibition through ferritin regulation.

The massive accumulation of lipid peroxidation products is a key factor in ferroptosis-mediated damage. Normally, lipid peroxides are eliminated by the antioxidant system centered on the SLC7A11/GPX4 axis. GPX4 converts lipid hydroperoxides into harmless alcohols, while SLC7A11 is responsible for cellular GSH synthesis and cystine uptake (Lee et al., 2021). In our study, cisplatin exposure markedly reduced SLC7A11 and GPX4 expression, depleted GSH, and increased ROS and MDA levels, indicating severe impairment of the intracellular antioxidant defense system. These biochemical alterations were accompanied by typical mitochondrial morphological abnormalities, including loss of cristae, mitochondrial shrinkage, rounding, and membrane rupture, all of which are consistent with ferroptotic injury. FN significantly reversed these changes in both HK-2 cells and AKI mice. In addition, FN improved cell viability in cisplatin- or RSL3-treated HK-2 cells, restored SLC7A11 and GPX4 expression, increased renal GSH levels, and reduced ROS and MDA accumulation. Together, these results indicate that FN suppresses cisplatin-induced ferroptosis at least in part by restoring the SLC7A11/GPX4 antioxidant system and limiting lipid peroxidation.

NRF2 is a master regulator of the cellular antioxidant response and has emerged as a key suppressor of ferroptosis. Under oxidative stress, NRF2 dissociates from KEAP1, translocates to the nucleus, and activates antioxidant response element-dependent transcription of multiple cytoprotective genes, including HO-1, SLC7A11, and GPX4-related antioxidant programs (Renaud et al., 2019). Through these downstream targets, NRF2 helps maintain redox homeostasis, detoxify ROS, and limit ferroptotic damage. HO-1, a classic NRF2 target, is also involved in heme degradation, iron handling, and oxidative stress responses, although its role in ferroptosis may depend on the cellular context (Yang et al., 2022). The expression of NRF2 and HO-1 proteins is crucial for kidney protection, as evidenced by their validated importance in various disease models (Lin et al., 2023). In the present study, cisplatin markedly reduced NRF2 and HO-1 protein levels in HK-2 cells, whereas FN restored their expression. These findings suggest that activation of NRF2/HO-1 signaling may be an important mechanism by which FN enhances antioxidant defense and suppresses ferroptosis in cisplatin-induced AKI. To further verify the role of NRF2, we performed loss-of-function experiments *in vitro* and *in vivo*. Knockdown of NRF2 in HK-2 cells weakened the inhibitory effect of FN on cisplatin-induced ferroptosis and reversed its regulation of FTH, FTL, SLC7A11, and GPX4. Similarly, pharmacological inhibition of NRF2 with ML385 in mice diminished the protective effects of FN on renal function, kidney injury markers, and the expression of NRF2, HO-1, and GPX4.

Increasing evidence indicates that PI3K/AKT acts upstream of NRF2 and promotes its activation under stress conditions. Mechanistically, PI3K/AKT signaling facilitates NRF2 stabilization and nuclear translocation, thereby enhancing transcription of antioxidant and anti-ferroptotic genes (Li et al., 2025). This signaling axis has been implicated in ferroptosis regulation in several disease models, including sepsis-associated liver injury and atherosclerosis (Wang J. et al., 2025; Yin et al., 2025). Mechanistic studies have demonstrated that PI3K/AKT promotes the dissociation of NRF2 from KEAP1 and its nuclear translocation via phosphorylation, thereby enhancing its transcriptional activity. The use of PI3K inhibitors significantly attenuates this effect, confirming that PI3K/AKT acts upstream of NRF2.

The role of the PI3K/AKT/NRF2 pathway in cisplatin-induced AKI has been preliminarily explored. In cisplatin-induced AKI, sustained oxidative stress leads to a transient increase in NRF2 levels; however, prolonged stress results in NRF2 depletion (Liu et al., 2025). Continuous activation of PI3K-AKT contributes to the maintenance of NRF2 levels (Lang et al., 2025; Li et al., 2025). One study reported that naringin alleviates cisplatin-induced renal injury by inhibiting apoptosis through the regulation of the PI3K/AKT/NRF2 signaling pathway (Sadhukhan et al., 2018). In non-renal injury contexts, multiple studies have shown that various small molecules or metabolites can inhibit ferroptosis by activating PI3K/AKT and subsequent NRF2 activity (Liu et al., 2021; Hu et al., 2025; Hu et al., 2025). Consistent with this concept, we observed that cisplatin reduced the phosphorylation of PI3K and AKT, whereas FN significantly restored their activation. Importantly, pharmacological inhibition of PI3K with LY294002 attenuated the effects of FN on NRF2 and GPX4, indicating that PI3K/AKT signaling is involved upstream of the NRF2-mediated anti-ferroptotic response.

Taken together, these results support a model in which FN alleviates cisplatin-induced AKI by activating the PI3K/AKT pathway, promoting NRF2/HO-1 signaling, restoring the SLC7A11/GPX4 antioxidant defense system, and ultimately suppressing ferroptosis.

To the best of our knowledge, this study provides new evidence that FN protects against cisplatin-induced AKI through inhibition of ferroptosis involving the PI3K/AKT/NRF2 signaling axis. These findings extend previous observations on the antioxidant and anti-inflammatory actions of FN and suggest that modulation of ferroptosis is an additional mechanism underlying its renoprotective effects.

However, several limitations should be acknowledged. First, many measurements were performed using whole-kidney tissue. Because cisplatin-induced renal injury is spatially heterogeneous, analyses based on bulk tissue may not fully reflect molecular changes occurring specifically in injured tubular segments. Future studies using cell-type-resolved approaches, such as laser microdissection, segment-specific isolation, or single-cell transcriptomic analysis, would provide a more refined understanding of ferroptosis in cisplatin nephrotoxicity. Second, although pharmacological inhibition and genetic interference supported the involvement of the PI3K/AKT/NRF2 axis, the direct molecular target of FN was not identified. Future studies using proteome-wide target screening, such as human proteome microarrays and surface plasmon resonance, may help clarify the direct binding partners of FN (Xuan et al., 2025). Third, FN has poor water solubility and limited oral bioavailability, with substantial plasma protein binding reported *in vivo* (Singh et al., 2011; Luo et al., 2018). Various studies have focused on developing delivery systems (e.g., self-microemulsifying drug delivery systems, nanoparticles) to enhance the solubility and bioavailability of FN (Zou et al., 2024; Liu et al., 2025). In our study, FN demonstrated a notable protective effect against cisplatin-induced ferroptosis in both *in vitro* and *in vivo* experiments. However, multiple studies have also reported that FN exerts distinct effects on immune cells and tumor cells (Zhou et al., 2023; Zhang et al., 2024; Zheng et al., 2024; Liu et al., 2025). Therefore, in the *in vivo* context characterized by low bioavailability, improving the targeting of FN for therapeutic purposes may represent a key focus of future research.

## 5 Conclusion

In summary, our study demonstrates that FN alleviates cisplatin-induced AKI by suppressing ferroptosis. FN restored antioxidant defense, reduced lipid peroxidation, improved mitochondrial integrity, and ameliorated iron metabolism-related disturbances, accompanied by upregulation of SLC7A11 and GPX4. Mechanistically, these effects were associated with activation of the PI3K/AKT pathway and subsequent enhancement of NRF2/HO-1 signaling. These findings suggest that FN may serve as a potential therapeutic candidate for cisplatin-induced AKI by targeting ferroptosis through the PI3K/AKT/NRF2 axis.

## Data Availability

The raw data supporting the conclusions of this article will be made available by the authors, without undue reservation.
